# Prevalence of long-acting reversible contraceptive methods utilization and associated factors among counseled mothers in immediate postpartum period at Jimma University medical center, Ethiopia

**DOI:** 10.1186/s40834-022-00184-x

**Published:** 2022-09-02

**Authors:** Wariyo D. Arero, Woubishet G. Teka, Habtemu J. Hebo, Terefe Woyo, Belay Amare

**Affiliations:** 1 Department of Gynecology and Obstetrics, Medical Science Faculty, Institute of Health, Jimma University, P.O.Box 378, Jimma, Ethiopia; 2 Department of Epidemiology, Public Health Faculty, Institute of Health, Jimma University, P.O.Box 378, Jimma, Ethiopia; 3 Department of Midwifery, College of Medicine and Health Sciences, Hawassa University, P.O.Box 1560, Hawassa, Ethiopia

**Keywords:** Long-acting reversible contraceptive methods, Prevalence, Associated factors, Immediate postpartum period, Ethiopia

## Abstract

**Background:**

Postpartum family planning is defined as the prevention of unintended pregnancy and closely spaced pregnancies through the first twelve months following childbirth. The immediate postpartum period is particularly favorable time to provide long-acting reversible contraception methods; and postpartum provision is safe and effective. Despite the advantages of long acting reversible contraception methods, they may be infrequently used in Ethiopia.

**Objective:**

This study assessed the prevalence and associated factors of long-acting reversible contraceptive methods utilization among counseled mothers in immediate postpartum period.

**Methods:**

A cross-sectional study was conducted on 393 women who gave birth at Jimma University Medical Centre from 12 November 2016 to 21 January 2017, Ethiopia. Data were collected by face-to-face interview using pre-tested structured questionnaire and by record reviewing using data compiling form; and analyzed using SPSS version 20. Logistic regression was used to identify associated factors for long acting contraceptive methods use. *P*-value less than 0.05 at 95% confidence level was taken as significance level.

**Results:**

Prevalence of reversible long acting contraceptive methods utilization among immediate postpartum mothers was 53.2% (209/393) and more than three-fourths (78.0%) of participants used implanon. The most common reported reason for not using reversible long acting contraceptive methods was preference of other contraceptive methods like short acting contraceptives (25.5%). Having more than four alive kids (AOR 2.6, 95% CI: 1.15,5.95), high monthly income (≥1000 ETB) (AOR 2.4, 95% CI: 1.08,7.20), planning to delay next pregnancy by more than 2 years (AOR 4.0, 95% CI: 1.60,9.28), mothers with no fertility desire (AOR 2.0, 95% CI: 1.12,3.15), prior use of reversible long acting contraceptive methods (AOR 3.0, 95% CI: 1.30,7.20) and receiving counseling during antenatal care follow-up and before delivery (AOR 2.0, 95% CI: 1.01, 4.73) were associated with immediate postpartum reversible long acting contraceptive methods use.

**Conclusion and recommendations:**

Although the prevalence of reversible long acting contraceptive methods utilization in immediate postpartum was high, counseling mothers during ANC follow-up and before delivery can further increase its utilization. Therefore, the need for providing counseling during ANC follow up and before delivery to increase utilization of immediate postpartum reversible long acting contraceptive methods use is emphasized.

## Background

Globally, the contribution of unintended pregnancy to maternal morbidity and mortality is significant [1]. Modern contraception is highly effective in preventing unintended pregnancy and reducing maternal mortality [2].

Reasons for unmet need are lack of services, limited choices, social disapproval, partner’s opposition, side effects and lack of knowledge about contraceptive options and their use [3–5]. Majority of women and girls with an unmet need for family planning are those who have recently given birth [6]. These vary by population and individual and made postpartum family planning programs difficult to design and administer [7].

Women are at risk of an unintended pregnancy in the period immediately after delivery [8]. Between 40 and 57% of women reported having unprotected intercourse before the routine 6-week postpartum visit [8–10]. Currently, WHO and USAID recommend the minimal live birth interval of 2 years to reduce the risks of abortion, miscarriage and still births [11, 12].

Postpartum contraceptive utilization is a primary strategy for reducing unintended pregnancy and optimizing birth spacing [13]. Long-acting reversible contraception methods are the most effective method of modern contraception [14]. LARC method is user-independent and once the device is inserted, the woman does not need any action to support ongoing effective utilization of the contraceptive [14]. It is more effective in preventing unintended pregnancy [15, 16]; has higher continuation rates than shorter-acting methods [16] and the return of fertility is rapid when removed [14, 16]. ACOG guidelines revised in 2012 advises that adolescents at high risk of unintended pregnancy should be encouraged to consider LARC methods as a contraceptive option [17]. WHO also supports the utilization of LARC methods for women of all ages [18]. LARC methods could prevent one in every three maternal deaths that causes related to pregnancy [19]. Immediate postpartum LARC methods insertion is recommended as best practices [20, 21]. Despite the higher expulsion rate, cost-benefit analysis data strongly suggest the superiority of immediate placement in the reduction of unintended pregnancy [22]. The hospital setting facilitates the availability of the contraception for the patient and the healthcare provider to motivate women for utilization of LARCs [8]. Ideally, women should be counseled prenatally about immediate postpartum LARC methods option for enabling informed decision making [21].

Ethiopia is the second most populous country in Africa with high maternal mortality ratio of 412 per 100,000 live births [12]. The Ethiopian government also set the goal to achieve a total fertility rate (TFR) of 2.1 by 2016 [23]. The government targeted 55% contraceptive prevalence rate by the year 2020, and 35% was expected to be LARC methods [23, 24]. From the statistics of EDHS 2016, LARC methods utilization was 8% for implant and 2% for IUD among married women whereas 11% for implant and 1% for IUD among sexually active unmarried women [12].

Although immediate postpartum is the best opportunity for LARC methods insertion, studies that documented LARC methods utilization and associated factors are very limited in Ethiopia. So, this study assessed prevalence and associated factors of LARC methods utilization among immediate postpartum mothers at one tertiary level teaching hospital in Jimma, Ethiopia. The findings of this study will have significant contribution at establishing strategic plans for local policy makers and NGOs working in the area.

**Fig. 1:**
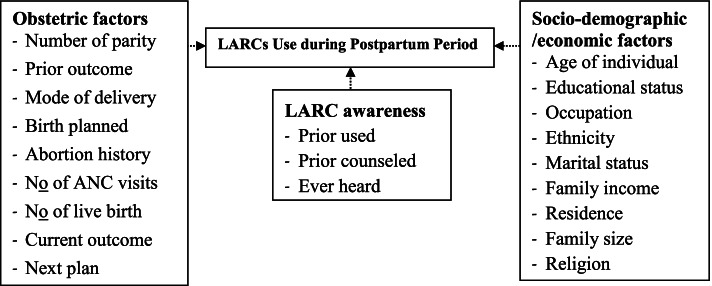
Conceptual framework on factors influencing LARC use in immediate postpartum period (prepared after related literature review)

Conceptual framework (Fig. 1).

## Methods and materials

### Study area and period

The study was conducted in Jimma University Medical Center (JUMC) at maternity ward from November 12, 2016 - January 21, 2017. JUMC is located in Jimma town at around 352 Km Southwest of Addis Ababa. It is the only teaching and referral hospital in the Southwestern part of the country, providing services for approximately 15 million people living in Jimma zone and Southwest Ethiopia. It is also serving as a clinical post graduate specialty teaching hospital for Obstetrics and Gynecology, Internal Medicine, Pediatrics and Child Health since 2005 and for Ophthalmology and Surgery since 2007. Department of Obstetrics and Gynecology has MCH unit, OPD, Family Planning unit, Referral unit, Gynecology and Maternity wards. The Maternity, Labor and Delivery ward has 60 beds in addition to seven first stage beds and 4 s stage couches. Services are provided by midwifes, medical interns and resident physicians and consultant Obstetricians and Gynecologists.

### Study design

Institution based cross-sectional study design was used.

#### Study population

All immediate postpartum mothers counseled for LARCs utilization during the study period were the study population.

#### Inclusion criteria

All immediate postpartum mothers who counseled for LARCs utilization during the study period.

#### Exclusion criteria

Mothers with puerperal sepsis, chorioamnionitis, deep venous thrombosis (DVT), congestive heart failure (CHF), severe liver disease, and previous breast cancer.

#### Sample size determination and sampling technique

The required sample size was determined by using single population proportion formula considering 36.7% prevalence (taken from community based study) [25], 5% level of significance, 5% margin of error and 10% non-response rate.

$$\mathrm{n}={\left( Z\alpha /2\right)}^2\ \mathrm{P}\left(1-\mathrm{P}\right)={(1.96)}^2\ (0.367)\ \left(1\_0.367\right)=357$$

$${\mathrm{d}}^2={(0.05)}^2$$

$${\mathrm{n}}_{\mathrm{f}}=357+10\%\ast 357=357+36=393$$

Convenience consecutive sampling technique was used. Beginning from the first date of data collection, all postpartum mothers who were candidate and counseled for LARC methods use were involved till the desired sample size was reached.

### Study variables

#### Dependent variable

Long acting reversible contraceptive methods utilization (Implanon, Jadle and IUDs) at immediate postpartum period.

#### Independent variables

Socio-demographic/economic variables (age, marital status, educational level, religion, ethnicity, occupation and residence, income, family size and husband support). Reproductive history of women (parity, number of live birth, prior outcome, mode of delivery, current birth outcome, and previous history of LARC methods use) and Prior LARC awareness (Prior used,Prior counseled and Ever heard).

### Data collection process

Data were collected by face-to-face interview using pre-tested structured questionnaire and by record reviewing using data compiling form. The questionnaire was developed according to objective of the study after reviewing different literature relevant to the study. The data collectors were two midwives working at maternity ward and one resident physician assigned to family planning unit who were also counseling about family planning. Data collectors and supervisors were briefed about the objectives of the study and the data collection tool by the principal investigator. The principal investigator and supervisors closely supervised the overall activities of the data collection on daily basis to insure the completeness of the questionnaire, to give further clarification and support for data collectors.

### Data quality management

The questionnaire was originally prepared in English and then translated to local languages (Afan Oromo and Amharic) and back translated to English to check for consistency of translation. The tool was pre-tested on 5% of the sample before actual data collection out of the selected health facility and necessary modifications were made based on nature of gaps identified. The midwives who collected the data were briefed on objective of the study, contents of the tool and how to approach participants for interview. On site supervision was carried out every day during the whole data collection period. At the end of each day, filled questionnaires were reviewed for completeness and consistency by supervisor and principal investigator. The data were cleaned and explored before analysis.

### Operational definitions

Immediate postpartum period is the time duration the women stayed in hospital before discharge after delivery of the baby [8].

Long-acting revisable contraception methods are contraception that prevent pregnancy ranging from 3 to 12 years [12].

### Data analysis

Data were entered into Epi Data Version 3.1, cleaned and analyzed using SPSS version 20. A descriptive analysis was carried out for each variable. Bivariate logistic regression was performed for independent variables that have adequate cell count to identify candidate variables for the multivariable logistic regression. Variables with *p*-value < 0.25 in bivariate analysis were entered into multivariable logistic regression model to determine independent effect of each covariate. Multicolinearity was assessed in linear regression with variance inflation factor (VIF) and none was found. Interaction was also assessed with Breslow-Day test and none was found. Model fitness was assessed by Hosmer-Lemeshow test and percentage of correct classification. In multivariable regression, association was analyzed at confidence level of 95% with their respective adjusted odds ratio and *p*-value of < 0.05.

## Result

### Socio- demographic/economic characteristics

Out of 393 mothers participated in the study, 41.7% of them were in the range of 25–29 years age group. The mean age value of these participants was 27 years. The majorities of participants were Muslims (239, [60.8%]), and Oromo (251, [63.9%]). Nearly all were married (373, [94.9%]), and near to one-third (31.6%) were housewives. Two third (63.1%) attended formal education of different levels and nearly half (185, [47.1%]) had monthly income between 1000 and 2500 ETB. The majorities (219, [55.7%]) of mothers were from rural (Table 1).

**Table 1:** Distribution of socio- demographic/economic characteristics of immediate postpartum mothers at JUMC, Nov 12, 2016 - Jan 21, 2017 (*n* = 393)

Variables		Number	%
**Age of mother**	15–19	24	6.1
	20–24	91	23.2
	25–29	164	41.7
	30–34	80	20.4
	35+	34	8.7
**Residence**	Rural	219	55.7
	Urban	174	44.3
**Religion**	Muslim	239	60.8
	Orthodox	81	20.6
	Protestants	60	15.3
	Catholics	11	2.8
	Others	2	0.5
**Ethnicity**	Oromo	251	63.9
	Amhara	55	14.0
	Dawro	32	8.1
	Gurage	30	7.6
	Others	25	6.4
**Marital status**	Married	373	94.9
	Single	9	2.3
	Divorced	7	1.8
	Widowed	4	1.0
**Educational status**	Can’t read and write	112	28.5
	Only read and write	33	8.4
	Primary school [1–8]	105	26.7
	High school [9–12]	87	22.1
	College or university	56	14.2
**Occupational status**	House wife	124	31.6
	Farmer	88	22.4
	Merchant	76	19.3
	Gov’t employee	64	16.3
	Private worker	19	4.8
	Student	17	4.3
	Other	5	1.3
**Monthly income (ETB)**	< 1000	108	27.5
	1001–2500	185	47.1
	> 2500	100	25.4

### Reproductive characteristics

Close to two-thirds (257, [65.4%]) of mothers were between para 2 and 4; almost all (98.7%) of participants have at least one alive kid. About 30.8% of mothers had two children; and the mean number of alive children of participants was 2.59. Nearly one-third (118, [30.0%]) of the current births were not planned for a time. Just two-thirds (66.2%) of the respondent had a plan to have children in the future. For those mothers who had a plan to have a child in the future, over three-fourths (200, [76.9%]) want to have a child after 2 years (Table 2).

**Table 2:** Reproductive information of mothers in immediate postpartum, JUMC, Nov 12, 2016 - Jan 21, 2017

Variables		Number	%
**Number of parity**	1	84	21.4
	2–4	257	65.4
	5+	52	13.2
**Mode of delivery**	SVD	212	53.9
	Assisted breech	12	3.1
	Instrumental	40	10.2
	Cesarean section	129	32.8
**Current birth outcome**	Alive	364	92.6
	Dead	29	9.4
**Number of alive kids**	1	91	23.5
	2–4	254	65.5
	5+	43	11.0
**Need more babies**	Yes	260	66.2
	No	102	26.0
	Yet not decided	31	7.9
**Next delivery plan within two years (*n*** **= 260)**	Yes	60	23.1
	No	200	76.9

### Awareness towards LARC, ANC follow up and prior LARC utilization history

Over three-fourths (309, [78.6%]) of the study participants ever heard about LARC methods from different sources. The main source of information was health workers (247, [79.9%]). Nearly 9 in ten (352, [89.6%]) of the study participant had at least one antenatal care visit (ANC) during the current last pregnancy. Only 106 (27%) had received counseling service on LARC methods during ANC visit. Ninety-two (23.4%) had previously used LARC method.

Out of these, 76(82.6%) used Implanon, 10(10.9%) used Jadelle/Sino implant and 6(6.5%) used IUD. Majorities of participants discontinued LARC because of the desire for pregnancy (Table 3).

**Table 3:** Awareness and utilization of LARC among mothers in immediate postpartum period, in JUMC, from Nov 12, 2016 - Jan 21, 2017

Characteristics		Frequency	%
**Ever heard about LARCs**	Yes	309	78.6
	No	84	21.4
**Ever counseled** **(ANC, labor, FP clinics)**	Yes	192	48.8
	No	201	51.2
**Time of counseled** ***N*** **= 192**	During ANC visit	106	55.2
	FP clinics	53	27.6
	During delivery	33	17.2
**Method previously used** ***N*** **= 92**	Implanon	76	82.6
	Sino-implant/Jadelle	10	10.9
	IUCD	6	6.5
**Reason for discontinuation**	Desire of pregnancy	66	71.7
	Side effects	14	15.2
	Influence by others	5	5.4
	Religious prohibition	3	3.3
	Others	4	4.3

### Prevalence of LARC methods utilization

**Fig. 2:**
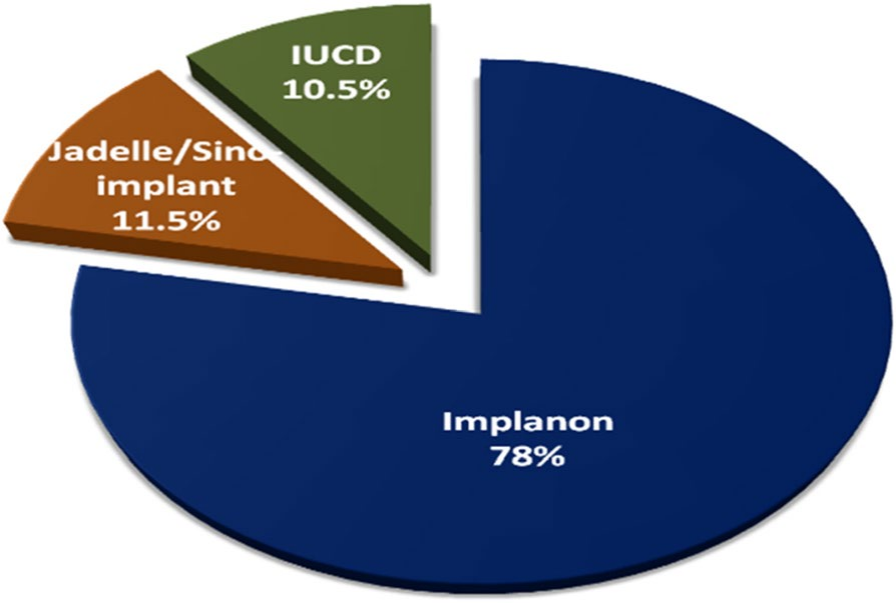
Pie chart showing current type of LARC methods used among immediate postpartum mothers during study period, in JUMC, from Nov 12, 2016 - Jan 21, 2017

**Fig. 3:**
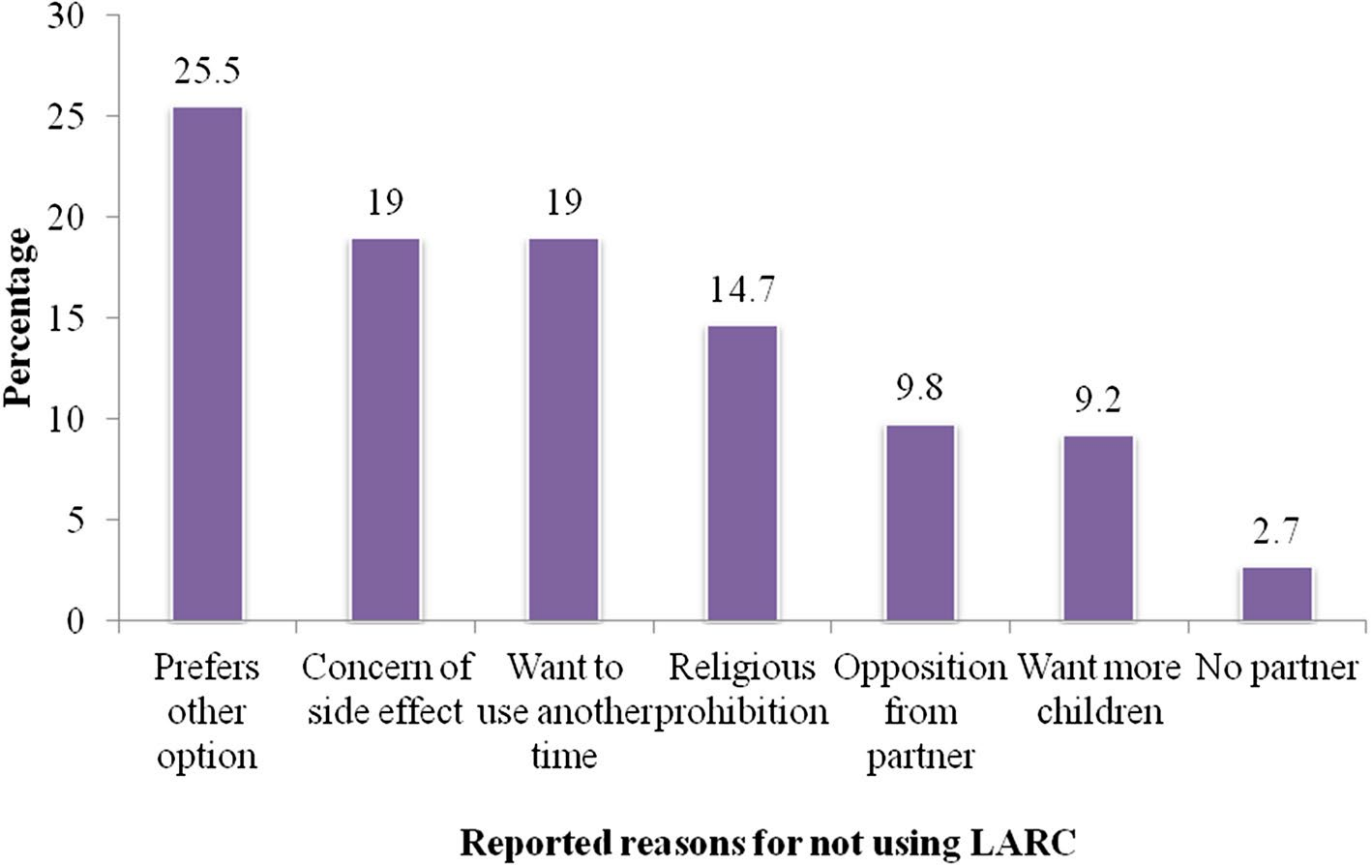
Percentage distribution of reasons of respondents not using LARC methods

The prevalence of current LARC use was (209, [53.2%]) (95% CI: 48.2–58.1). Over three-fourths (78%) used Implanon, 11.5% used Jadelle/Sino Implant and 10.5% used IUD (Fig. 2). Various reasons were reported for not using LARC methods during study period. The commonest reported reason was preference of other form of contraceptive methods (25.5%) and others were fear of side effects, religious prohibition, want to use LARC methods other time, opposition from partner and want to have more children (Fig. 3).

### Factors associated with LARC use

Bivariate and multivariable logistic regression analyses were done to identify factors associated with LARC method use. The results of these analyses showed that being counseled at ANC, monthly income greater than 1000 ETB, family size more than four, completed family size, having plan to delay next pregnancy beyond 2 years and prior use of LARC have increased chance of current immediate postpartum use of LARC methods.

Mothers who had monthly family income of 1000 ETB or more were 2.4 times more (AOR = 2.4, 95% CI: 1.08–7.20) likely to use LARC methods compared to mothers with monthly family income less than 1000 ETB. Mothers who had more than four alive kids were 2.6 times more (AOR = 2.6, 95% CI: 1.15–5.95) likely to use LARCs than mothers who had none or only one child. Women who completed family size (no desire for future fertility) were approximately two times more (AOR = 2.0, 95%CI: 1.12–3.15) likely to use LARC methods compared with women who need more children. Mothers who planned to extend next birth beyond 2 years were nearly four times more (AOR = 4.0, 95%CI: 1.60–9.28) likely to use LARC methods than mothers who planned next birth within next 2 years. Mothers who previously used LARC were three fold more (AOR = 3.0, 95% CI: 1.30–7.20) likely to use LARCs than mother who never tried it. Most importantly, mothers counseled for LARC during ANC visits were two fold higher (AOR = 2.0, 95%CI: 1.01–4.73) used LARC methods at immediate postpartum than mother who were not counseled (Table 4).

**Table 4:** Multivariable logistic regression result of current LARC use among mothers in immediate postpartum period, JUMC, Nov 12, 2016 to Jan 21, 2017

Respondents’ characteristics		Current LARC use		COR (95%CI)	AOR (95%CI)
		Yes	No		
Maternal age	15–24	60	55	1^¥^	
	25–34	127	117	1.0(0.64, 1.55)	1.1(0.56, 2.16)
	35+	22	12	1.7(0.76, 3.71)	1.2(0.34, 3.83)
Mode of delivery	Vaginal	132	132	1	
	Cesarean	77	52	1.5(1.0, 2.27)^*^	1.6(0.82, 3.02)
Current birth outcome	Dead	12	17	1	
	Alive	197	167	1.7(0.78, 3.60)	2.5(0.41, 15.35)
Number of Parity	1	47	37	1	
	2–4	125	132	0.7(0.45, 1.22)	0.19(0.03, 1.48)
	5+	37	15	1.9(1.0, 4.06) ^*^	0.50(0.11, 2.32)
Number of alive kids	≤ 1	46	45	1	
	2–4	129	125	1.0(0.63, 1.62)	0.8(0.51, 1.41)
	5+	31	12	2.5(1.16, 5.52) ^*^	2.6(1.15, 5.95) ^*^
Completed family size	No	146	146	1	
	Yes	68	34	2.0(1.24, 3.23) ^*^	1.9(1.12, 3.15) ^*^
Next delivery plan within two years	Yes	14	46	1	
	No	119	81	4.9(2.61, 9.20) ^*^	3.8(1.60, 9.28) ^*^
Ever heard of LARC	No	24	60	1	
	Yes	185	124	3.7(2.21, 6.31) ^*^	1.2(0.47, 3.11)
Counseled at ANC	No	89	157	1	
	Yes	67	39	3.0(2.00, 4.60) ^*^	2.1(1.01, 4.73) ^*^
Previous LARC use	No	46.2	53.8	1	
	Yes	70	22	3.7(2.18, 6.30) ^*^	3.1(1.30, 7.20) ^*^
Monthly income	< 1000 ETB	39	67	1	
	> 1000 ETB	170	115	2.6(1.65, 4.14) ^*^	2.4(1.08, 4.73) ^*^

^*^ *COR significant at p-value < 0.05;* 1 ^¥^ logical reference; ^*^AOR significant at p-value < 0.05

## Discussion

We assessed the prevalence and associated factors for utilization of LARC methods among immediate postpartum mothers at JUMC, Southwest Ethiopia. The prevalence of current immediate postpartum LARC use was 53.2%. Over three-fourths (78%) used implanon followed by Jadelle/Sino Implant (11.5%) and IUD (10.5%). The commonest reported reason for not using LARC was preference of other contraceptive methods like short acting contraception. Being counseled at ANC, monthly income greater than 1000 ETB, family size more than four, completed family size, having plan to delay next pregnancy beyond 2 years and prior use of LARC have increased chance of current immediate postpartum use of LARC methods.

The prevalence estimated in our study was much higher than national estimates where only 10% of women used LARC as reported in EDHS 2016 [12]. This could be because of the fact that national estimate was for all reproductive age group women whether married or unmarried. But, our study was limited to specific group of women (immediate postpartum women) who are more likely to accept LARCs. The proportion of women who used IUD in our study was much lower than 21.9% prevalence of immediate postpartum IUD use reported by facility based cross-sectional study conducted in Southern Ethiopia [26]. Though there was similar proportion of ANC use (89.6% vs 84.2%), there was significantly lower proportion of counseling for LARCs at ANC (27% vs 72.3%) and all were counseled at postpartum in our study. Hence, the lower percentage of IUD use could be because of the combination of lower proportion of counseling at ANC and availability of alternative LARC options in our study but, only IUD in the case of study conducted in Southern Ethiopia [26]. However, it was similar to 12.4% prevalence of immediate IUD use reported by facility based cross-sectional study conducted in Bale zone, Southeast Ethiopia which reported similar proportion (87.6%) of ANC use [27].

Our finding was also higher than 36.7% [25] prevalence of LARC use in the extended postpartum period (42 days to 1 year) reported by community based cross-sectional study conducted in Southern Ethiopia. But the proportion of women counseled for LARC was significantly lower in our study (27% versus 51.5%). This indicates that LARC acceptance is better at immediate postpartum and mothers may change their mind and reject LARC offer at extended postpartum even if they were willing to use it at immediate postpartum.

Our finding was also higher than 22.9% [28] and 16% [29] prevalence of LARC use among family planning attendees of public health facilities in Jimma town, Southern Ethiopia. It was also higher than 38% [30], 29.1% [31], 37.7% [32], 25.2% [33], 28.3% [34], 30.3% [35] and 23.8% [36] prevalence of LARC use among family planning clients reported by community based studies conducted in different parts of Ethiopia. It was also higher than 33.7% [37], 16.4% [38], 33.7% [39], 16.3% [40], 28% [41], 17.6% [42] and 9.24% [43] prevalence of LARC use among family planning clients reported by facility based cross-sectional studies conducted in different parts of Ethiopia. The finding was also higher than 37.4% [44] prevalence of LARC use among HIV positive family planning attendees of public health facilities in Bahir Dar town, Northern Ethiopia. We also calculated 23.84% pooled prevalence from eighteen facility or community based cross-sectional studies conducted in different corners of Ethiopia [25, 28–44] and found that our finding was higher than pooled prevalence.

Regarding type of LARC use, birth control Implant was the method used by almost 9 out of ten mothers. This finding was in line with findings of most previous studies conducted in different parts of Ethiopia where Implant was used by at least three-fourths of mothers [25, 28–34, 36, 38, 40, 41, 43–45] and higher than findings of some studies [35, 37, 39]. This could be because of convenience and privacy as implants are inserted under the skin into the upper arm area whereas IUDs are inserted into the uterus. Thus, women may think that it’s painful while inserting IUDs into uterus especially during immediate postpartum and/or during sexual intercourse and/or while walking. They may also think that it can cause damage to the uterus. In facility based cross-sectional study conducted in Bale zone, Ethiopia, one-third of study participants agreed and only one-fifth disagreed that insertion and removal of IUD is highly painful. In the same study, more than one-third (37.6%) agreed that insertion of IUD causes loss of privacy and 41.6% agreed that IUDs may impair future fertility [27]. In another community based study cross-sectional study, nearly one-third (31%) of study participants disagreed with the statement “insertion of intrauterine contraceptive devices does not lead to loss of privacy”. Similarly, nearly half (46%) disagreed with the statement “using intrauterine contraceptive devices does not restrict normal activities” [36].

In this study, counseling at ANC was significantly associated with immediate postpartum LARC utilization. Studies conducted in different corners of Ethiopia reported that women counseled at ANC and/or during delivery and/or postpartum and/or received postnatal care were more likely to use LARC [25, 26, 34]. This could because women who received postnatal care were likely to be counseled for LARC and counseling increases women’s knowledge of LARC including its advantage and disadvantage and clears misconceptions increasing chance of LARC use. Previous studies have reported that women who heard [26], had awareness [40], had information [33] about LARC, had moderate or high knowledge of LARC [31, 37, 39, 45] or previously used it [25, 28, 41] were more likely to utilize it now. Prior use of LARC was also positively associated with current use of LARC in our study which was in line with literature. Studies have also reported that women with misconception [31, 36] and who heard myths [44] were less likely to use LARC. On the other hand, positive/supportive attitude towards LARC [31, 38], not hearing myths [29], health professionals being source of information [45] and discussion of LARC with providers [29] were positively associated LARC use. Similarly, maternal literacy was also positively associated with LARC use [25, 27, 28, 30–32, 34, 37, 39, 40, 42] because education is likely to enhance women’s autonomy and confidence to make decision regarding their own health and demand higher quality of life. The association between prior use of LARC and current acceptance is an indication of knowledge influence.

In general, counseling builds knowledge of LARC, clears misconception and myths about LARC, develops positive/supportive attitude and finally leads to increased use of LARC. However, although counseling for postpartum family planning is also acceptable during early labor and immediately postpartum, it should optimally begin during ANC according to WHO recommendations [46] and it is the ideal time to counsel women. In this study, however, only 27% of participants were counseled at ANC follow up though all were counseled at immediate postpartum. This finding indicates the importance of integrating counseling for post-partum family planning into ANC and/or early labor and/or the immediate postpartum period to increase postpartum LARC utilization.

### Strength and limitations of study

Integrating counseling for post-partum family planning at immediate postpartum period increased postpartum LARC utilization.

This study was institution based and the respondents were immediate postpartum mothers (48 hours before hospital discharge) who were counseled for LARC. Therefore, the study findings may be not be generalized to all reproductive age women in the community. Counseling for contraception options was done after delivery but is better addressed before delivery especially for IUD to be inserted just post-placental delivery in either vaginal or cesarean delivery. Another limitation of this study was some mothers might have been discharged from the hospital before counseled and interviewed though we were actively looking for all postpartum mothers. The study was done in 2016–2017. In addition, convenience consecutive sampling technique is used which affects generalization of the study.

### Implications for research and policy

The findings of this study will be baseline data for future researchers and local policy makers working on the area of the study.

## Conclusion and recommendations

The advantages of postpartum contraceptive use for wellbeing of mother and child cannot be overemphasized. The prevalence of immediate postpartum LARC use was promising with implant the most preferred method by mothers. Counseling at ANC follow up, monthly income greater than 1000 ETB, family size more than four kids, completed family size (no future desire for fertility), having plan to delay next pregnancy beyond 2 years and prior use of LARC were important factors associated with increased chance of uptake of LARC methods at immediate postpartum period. This is the indication of importance of knowledge on LARC methods that clears misconceptions or myths and builds positive/supportive attitude, ultimately leading to increased utilization of LARC methods. Therefore, it is recommended to consider counseling for LARC methods in continuum starting at ANC follow-up through postpartum period.

## Data Availability

All datasets on which the conclusions of the paper rely were presented in the main manuscript.

## Abbreviations

ACOG: American College of Obstetricians and Gynecologists
ANC: Antenatal Care
CHF: Congestive heart failure
CBE: Community based education
DVT: Deep venous thrombosis
EDHS: Ethiopian Demographic and Health Survey
ETB: Ethiopian Birr
IUDs: Intra Uterine Devices
JUMC: Jimma University Medical Centre
LARC: Long reversible contraceptive
MCH: Maternal and child health
OPD: Outpatient department
PPFP: Postpartum family planning
TFR: Total fertility rate
USAID: United States Agency for International Development
WHO: World Health Organization
